# Supplementary material for: Single-cell perturbation prediction near experimental reproducibility with GENESIS
Source: bioRxiv. 2026 Nov 1:2026.10.30.763081. Preprint. [Version 1] doi: 10.64898/2026.10.30.763081 (PMC13734124; doi:10.64898/2026.10.30.763081)
Supplement: Supplementary file 1 [file NIHPP2026.10.30.763081v1-supplement-1.pdf]

bioRxiv preprint doi: https://doi.org/10.64898/2026.10.30.763081; this version posted November 1, 2026. The copyright holder for this preprint (which was not certified by peer review) is the author/funder, who has granted bioRxiv a license to display the preprint in perpetuity. It is made available under a CC-BY 4.0 International license.

Supplementary Methods

Training optimization steps

Training on the full Tahoe-100M biological-context split for five epochs required 256,205 optimizer steps. On the reduced dataset retaining 25% of perturbed cells and all controls (approximately 24.2 million cells), validation loss stopped improving after approximately 150,000 steps. We used this plateau to set a shorter training budget and derive a scaling rule for other dataset sizes.

Specifically, we fitted a power law through the full-data budget and the validation-based budget for the 25%-cells dataset:

$$\alpha = \frac{\log(150{,}000/256{,}205)}{\log(24.2/91.8)} \approx 0.40, \qquad T(N) = 256{,}205\left(\frac{N}{91.8 \times 10^6}\right)^{\alpha},$$

where N is the total number of training cells, including controls. PBMC models trained for five epochs (20,425 steps), taking approximately four hours on one NVIDIA H100 NVL.

Fast-training configuration.

The 27 repeated runs for the training-overlap experiments (Fig. 3b) and related data-efficiency analyses used 96 generated cells per observation and batches of 2,048 observed cells. The architecture, objective and other optimizer settings followed the default configuration.

Statistical baselines

We compared GENESIS with five statistical baselines (perturbation mean, context mean, linear ridge, additive and nearest neighbor), each fitted on the training conditions and gene panel of the corresponding analysis (the 2,000 panel genes unless stated otherwise). We index biological context by b, perturbation by p, and experimental replicate by r, where r in Tahoe-100M identifies a plate. Let Y_{bpr} and $\hat{Y}_{bpr}$ denote the true and predicted cell-expression distributions, and let $\mathbf{y}^{(i)}_{bpr}$ and $\hat{\mathbf{y}}^{(i)}_{bpr}$ denote individual observed and predicted cells, as counts on the panel genes. Following previous works[3,14], we normalize and log-transform each cell's counts:

$$\mathbf{y}^{(i)}_{\text{norm}} = \log\left(1 + 10^4\, \mathbf{y}^{(i)}/L^{(i)}\right),$$

where $L^{(i)}$ is the cell's full-transcriptome library size, defined as its total count over all measured genes. We write $\boldsymbol{\mu}_{bpr}$ for the mean of $\mathbf{y}^{(i)}_{\text{norm}}$ over a condition's cells and $\boldsymbol{\mu}^0_{br}$ for the same mean over its matched control cells. The observed transcriptomic response is

$$\mathbf{d}_{bpr} = \boldsymbol{\mu}_{bpr} - \boldsymbol{\mu}^0_{br}.$$

In Tahoe-100M, biological context denotes a cell line, perturbation denotes a compound–dose pair, and controls (DMSO) are matched by cell line and plate. In the PBMC cytokine atlas, biological context denotes a donor-cell-type combination and perturbation denotes a cytokine. Each donor occupies its own plate, so r is determined by the donor. Each donor-cytokine condition occupies a single well, and its controls pool cells of the corresponding cell type from the donor's six PBS wells.

Generating populations from pseudobulk vectors

To turn a predicted perturbed pseudobulk into a cell population, we subtract the observed control pseudobulk and add the resulting difference to individual control cells. This shifts the control population toward the predicted perturbed expression while incorporating variation from real cells,

bioRxiv preprint doi: https://doi.org/10.64898/2026.10.30.763081; this version posted November 1, 2026. The copyright holder for this preprint (which was not certified by peer review) is the author/funder, who has granted bioRxiv a license to display the preprint in perpetuity. It is made available under a CC-BY 4.0 International license.

allowing evaluation using energy distance and DEG overlap. In normalized log-expression space, each sampled control cell is shifted as

$$\hat{\mathbf{y}}^{(j)}_{\text{norm},bpr} = \max\left(\mathbf{y}^{0(j)}_{\text{norm},br} + \underbrace{\hat{\boldsymbol{\mu}}_{bpr} - \boldsymbol{\mu}^{0}_{br}}_{\text{pseudobulk } \Delta}, 0\right).$$

Context mean.

The context mean predicts the average expression observed across training perturbations in the target biological context. It captures the context's typical perturbed expression without distinguishing between perturbations. Mathematically,

$$\hat{\boldsymbol{\mu}}_{bpr} = \left\langle \boldsymbol{\mu}_{bp'r} \right\rangle_{p'},$$

where the average runs over the non-control training perturbations measured in context b on plate r. If no such conditions are available on the target plate, we average over all non-control training conditions of b across plates.

Perturbation mean.

The perturbation mean assumes that a perturbation induces a shared expression change across biological contexts. It averages the matched-control responses of training conditions containing that perturbation and adds the resulting response to the target context's control mean. Mathematically,

$$\bar{\mathbf{d}}_p = \frac{1}{|\mathcal{T}_p|} \sum_{(b',r') \in \mathcal{T}_p} \mathbf{d}_{b'pr'}, \qquad \hat{\boldsymbol{\mu}}_{bpr} = \boldsymbol{\mu}^{0}_{br} + \bar{\mathbf{d}}_p,$$

where $\mathcal{T}_p$ contains the eligible training context-plate conditions measured with perturbation p. The baseline therefore captures a shared perturbation response without modeling a response specific to the target context.

Additive model.

The additive model explains an expression response through separate contributions from biological context and perturbation. It allows contexts to differ in their overall response and perturbations to differ in their shared effects, without learning a context-perturbation interaction. Mathematically,

$$\hat{\mathbf{d}}_{bp} = \boldsymbol{a} + \boldsymbol{\alpha}_b + \boldsymbol{\beta}_p, \qquad \hat{\boldsymbol{\mu}}_{bpr} = \boldsymbol{\mu}^{0}_{br} + \hat{\mathbf{d}}_{bp}.$$

The effects are estimated independently for each gene and sequentially from the training responses. First, $\boldsymbol{a}$ is the mean response over all training conditions. Next, $\boldsymbol{\alpha}_b$ is the mean residual over conditions of context b. Finally, $\boldsymbol{\beta}_p$ is the mean remaining residual over conditions of perturbation p. This sequential procedure defines the effects without additional identifiability constraints.

Linear ridge regression.

The linear ridge regression baseline represents shared perturbation responses through a low-dimensional description of genes. It constrains the perturbation-mean responses to this gene representation, with regularization shrinking the fitted coefficients.

Following ref. 14, let D contain the perturbation-mean responses $\bar{\mathbf{d}}_p$ as columns, and let $\boldsymbol{\nu}$ be its row mean. Let G be the genes-by-100 matrix of gene scores from the first 100 principal components

bioRxiv preprint doi: https://doi.org/10.64898/2026.10.30.763081; this version posted November 1, 2026. The copyright holder for this preprint (which was not certified by peer review) is the author/funder, who has granted bioRxiv a license to display the preprint in perpetuity. It is made available under a CC-BY 4.0 International license.

of the training pseudobulk matrix, whose columns contain mean raw-count profiles centred within each condition. Let P be the identity matrix encoding perturbations as one-hot vectors.

We approximate

$$D \approx GKP^\top + \boldsymbol{\nu}\mathbf{1}^\top$$

using the regularized closed-form estimator of the reference implementation,

$$K = (G^\top G + \lambda I)^{-1} G^\top (D - \boldsymbol{\nu}\mathbf{1}^\top) P (P^\top P + \lambda I)^{-1}.$$

We fix $\lambda = 0.1$ to the reference implementation's value rather than selecting it on validation data. No gradient-based optimization is required.

Writing $\mathbf{e}_p$ for the one-hot vector selecting the relevant response column, the prediction is

$$\hat{\boldsymbol{\mu}}_{bpr} = \boldsymbol{\mu}^0_{br} + GK\mathbf{e}_p + \boldsymbol{\nu}.$$

The fitted perturbation response is shared across eligible biological contexts.

Nearest neighbor.

We introduce a nearest-neighbor baseline that predicts a perturbation's effect by copying its observed cells from a biological context with similar responses to other perturbations. For each training condition, we compute a response by subtracting the matched control mean from the perturbed mean after applying $\log(1+y)$ to individual cell counts. Responses measured on multiple plates are averaged in proportion to their perturbed cell counts, then projected onto 100 principal axes fitted to the training responses. We compare two contexts using their average cosine distance over shared training perturbations with nonzero projected responses. To predict a held-out pair (b, p), we select the closest eligible training context measured with p. Candidates must share at least one usable training perturbation with the target context. This procedure applies to both biological-context and perturbation generalization.

Hierarchical balanced stratified sampling

To construct reduced training sets with fewer experimental conditions while maintaining approximately balanced representation of the biological contexts and perturbations axes, as in Figure 3, we use a hierarchical balanced stratified sampling (HBSS) procedure. Unlike uniform sampling, which preserves imbalances in the original dataset, HBSS assigns higher selection probabilities to underrepresented combinations. For example, in the Tahoe-100M biological-context split, the five evaluation cell lines have substantially fewer training combinations than the other cell lines because most of their perturbation responses were withheld. Uniform sampling would preserve this imbalance, whereas HBSS increases their representation in the subsampled training set. Only perturbed combinations are sampled; all control cells remain in training.

Inclusion weights.

Let Ω be the N measured combinations (b, p, ℓ) of cell line b, compound p and dose ℓ, with plates pooled, and let $n = \mathrm{round}(fN)$. Starting from $Q_{bp\ell} = n/N$ on Ω and 0 elsewhere, iterative proportional fitting rescales Q along each axis in turn towards uniform sums ($n/|\mathscr{B}|$ per line, $n/|\mathscr{P}|$ per compound, $n/|\mathscr{L}|$ per dose), clipping every entry to at most 1 after each rescaling so that $Q_{bp\ell}$ remains an inclusion probability. Iteration stops once no entry changes by more than 10^{-4} in a sweep.

bioRxiv preprint doi: https://doi.org/10.64898/2026.10.30.763081; this version posted November 1, 2026. The copyright holder for this preprint (which was not certified by peer review) is the author/funder, who has granted bioRxiv a license to display the preprint in perpetuity. It is made available under a CC-BY 4.0 International license.

Quotas.

Each compound-dose pair receives an integer number of cell lines $q_{p\ell}$ by largest-remainder rounding of $\sum_b Q_{bp\ell}$, so that $\sum q_{p\ell} = n$; a quota exceeding the number of cell lines measured with the pair is capped there and the excess passed to pairs with unused cell lines.

Selection.

Within each pair, cell lines are drawn one at a time without replacement with probability proportional to $\max\{Q_{bp\ell} - \lambda c_{\phi(b)}, 0\}$, where $c_{\phi(b)}$ counts the lines already drawn from the tissue type of b for this pair and $\lambda = 0.5$; if every remaining score is zero, the next line is drawn uniformly. Because λ exceeds most weights, a second cell line from a tissue type is drawn only once every other tissue type has one, with a goal of increasing tissue type. .

Settings.

Tissue types are defined by the cell lines' organ annotations. The 5% and 25% selections were drawn independently (seed 0). In the PBMC atlas, every donor-cytokine combination is measured, and training combinations are uneven only because held-out donors contribute fewer cytokines; HBSS there balances donors and cytokines without a tissue term.

Biological program recovery with Hallmark GSEA

To assess whether GENESIS recovers biological programs under an alternative gene-set collection and scoring method, we complemented the PROGENy analysis with preranked GSEA[42] of the 50 MSigDB Hallmark gene sets (v2026.1[43]). GENESIS was trained on 15% of training conditions using a 5,750-gene panel covering 4,376 of 4,384 Hallmark genes; additive and nearest-neighbor baselines used the same training conditions. Evaluation covered 75 compounds at 5 μM across five held-out cell lines (375 conditions), each measured on at least two plates with at least 50 perturbed and 50 control cells per plate. GENESIS outperformed both baselines across gene sets, compounds and cell lines. Across gene sets, its mean Pearson correlation with measured responses was 0.32, approaching experimental reproducibility of 0.38 (Extended Data Fig. 13).

Plate-specific responses followed the pseudobulk and library-depth procedures in Methods. Genes were retained at a control detection fraction of at least 10%, averaged equally across plates. Predictions used the measured detection filter and depth adjustment, with additional source-line detection filtering for nearest neighbor. Genes were ranked by adjusted log-fold change, resolving ties by gene name, and scored using preranked GSEA (GSEApy[44]), with all 50 gene sets retained. Normalized enrichment scores were evaluated using Pearson correlations across gene sets, compounds and cell lines, following the cross-plate comparisons and cluster-bootstrap procedures described in Methods. For the compound measured on three plates, statistics were averaged over three complete analyses, each using a different plate pair.

Ablations

Conditioning covariates in Tahoe-100M.

GENESIS generates predictions conditional on all covariates available. To assess the contribution of different conditioning covariates, we trained four GENESIS models using progressively complete inputs: plate alone; plate and cell line; plate, compound and dose; or all four covariates. All four models were trained on the faster configuration (generated repeats per true cell $m = 96$) and were compared only with one another (Extended Data Fig. 15).

bioRxiv preprint doi: https://doi.org/10.64898/2026.10.30.763081; this version posted November 1, 2026. The copyright holder for this preprint (which was not certified by peer review) is the author/funder, who has granted bioRxiv a license to display the preprint in perpetuity. It is made available under a CC-BY 4.0 International license.

Energy score versus Gaussian-kernel MMD.

To test the effect of the training objective, we retrained GENESIS on the 25% training-cell subset used in Fig. 3a, replacing the energy score and variogram with a Gaussian-kernel MMD objective. MMD is commonly used to fit cell distributions[3,4]. With State's energy-distance kernel, MMD^2 equals energy distance[3]; we therefore used a Gaussian kernel to compare distinct objectives. The replacement loss was

$$S(P_\theta, y) = \tfrac{1}{2}\,\mathbb{E}\,k(X, X') - \mathbb{E}\,k(X, y), \qquad k(a,b) = \frac{1}{5}\sum_{s=1}^{5} \exp\left(-\frac{\|a-b\|_2^2}{2\sigma_s^2}\right),$$

where X, X' are independent model samples and $\sigma_s = 13.43 \times \{\frac{1}{4}, \frac{1}{2}, 1, 2, 4\}$. The base bandwidth was fixed before training as the median pairwise distance among 4,096 training cells in log(1+count) space. Averaged over observed cells, this strictly proper score equals $\frac{1}{2}\mathrm{MMD}^2$ up to a constant.

Each training step used seven conditions, with up to 256 observed and 256 generated cells per condition, weighted by each condition's training-cell count. We trained for 280,000 optimizer steps, compared with 278,120 for GENESIS. This condition-level objective used up to 1,792 generated cells per step; GENESIS's per-observed-cell objective used 458,752. Alongside the standard metrics, we evaluated both models using unbiased MMD^2 with a single Gaussian kernel, setting its bandwidth to the median pairwise distance in each comparison (Extended Data Fig. 16).

Effect of training settings

We assessed the performance cost of reducing the number of generated cells and the network size, and examined the contribution of the variogram term. Starting from the model of Fig. 2, we varied each setting separately while keeping the remaining training settings fixed.

Number of generated cells.

We evaluated whether fewer generated cells per observation could reduce training cost while retaining predictive performance. We tested $m \in \{16, 64, 256\}$, with $m = 256$ used in the main model. At the fixed minibatch size of 1,792 observed cells, these settings generate 28,672, 114,688 and 458,752 cells per step, respectively. All generated cells contributed to the energy score and variogram estimators. Five-epoch training times were approximately 4.3, 11.0 and 43.3 hours, respectively (Extended Data Fig. 14a).

Variogram weight.

We introduced the variogram term to emphasize gene-gene dependencies during training. To assess its contribution, we evaluated weights $\lambda \in \{0, 0.1, 0.3, 0.5, 1\}$, including the energy-score-only objective at $\lambda = 0$ and the main model's setting of $\lambda = 0.5$ (Extended Data Fig. 14b).

Network architecture.

We evaluated whether smaller networks could retain the performance of the main architecture. We compared zero hidden layers, one hidden layer (8,192 units) and the main model's two hidden layers (4,096 and 8,192 units), comprising 2.7, 27.0 and 55.3 million parameters, respectively. All networks received a 1,280-dimensional input comprising four 256-dimensional embeddings (cell line, compound, dose and plate) and a 256-dimensional noise vector. The output head was a linear map to 2,000 genes followed by ReLU, acting directly on the input in the zero-hidden-layer model. Five-epoch training times were approximately 16.8, 31.3 and 43.3 hours, respectively (Extended Data Fig. 14c).

bioRxiv preprint doi: https://doi.org/10.64898/2026.10.30.763081; this version posted November 1, 2026. The copyright holder for this preprint (which was not certified by peer review) is the author/funder, who has granted bioRxiv a license to display the preprint in perpetuity. It is made available under a CC-BY 4.0 International license.

Supplementary Tables

Supplementary Table 1 | Model performance relative to experimental reproducibility on Tahoe-100M biological-context generalization (Fig. 2a). Predictions were evaluated against observations from the other plate across 530 plate pairs. R is the mean model score divided by the mean experimental-repeat score across plates. $R = 1$ indicates equal mean scores; higher values are better for DEG overlap and Pearson Δ, and lower values are better for energy distance. Win rate is the fraction of cross-plate predictions that outperform the repeat, counting ties as half. Confidence intervals (95%) and two-sided tests account for clustering by combination, as described in Methods. The tests compare R with 1 and win rate with 0.5; stars indicate $p \geq 0.05$ for both. Methods are ranked by mean performance, with the best in bold and second best underlined.

Method	R (95% CI)	Win rate (95% CI)	p (R)	p (win)	Star
**Direction-matched DEG overlap**					
**GENESIS**	**0.966 [0.946–0.986]**	0.451 [0.424–0.479]	8.1×10^{-4}	6.8×10^{-4}	—
STATE	0.905 [0.869–0.943]	0.396 [0.364–0.428]	2×10^{-6}	5.7×10^{-10}	—
PerturbDiff	0.767 [0.729–0.808]	0.360 [0.329–0.392]	3.7×10^{-22}	6.9×10^{-17}	—
Nearest-neighbor	0.551 [0.530–0.572]	0.195 [0.169–0.220]	1.7×10^{-117}	7.7×10^{-83}	—
CellFlow	0.511 [0.480–0.544]	0.275 [0.239–0.310]	7.5×10^{-70}	7×10^{-32}	—
CtxMean	0.133 [0.123–0.143]	0.116 [0.095–0.137]	1.9×10^{-201}	2.1×10^{-137}	—
Additive	0.076 [0.069–0.085]	0.080 [0.060–0.099]	8×10^{-190}	1×10^{-169}	—
PertMean	0.024 [0.019–0.030]	0.055 [0.042–0.069]	1.1×10^{-122}	1×10^{-246}	—
LinRidge	0.017 [0.013–0.022]	0.055 [0.041–0.068]	3.3×10^{-107}	4.9×10^{-249}	—
**Pearson Δ**					
**GENESIS**	**0.995 [0.977–1.013]**	0.473 [0.440–0.505]	0.56	0.10	★
STATE	0.839 [0.810–0.868]	0.315 [0.279–0.351]	1.6×10^{-21}	8.6×10^{-22}	—
Additive	0.685 [0.642–0.731]	0.260 [0.223–0.298]	3.3×10^{-27}	1.5×10^{-31}	—
PerturbDiff	0.677 [0.649–0.706]	0.238 [0.203–0.273]	1×10^{-56}	8.6×10^{-41}	—
CtxMean	0.600 [0.573–0.629]	0.214 [0.181–0.247]	2.1×10^{-74}	4.7×10^{-51}	—
Nearest-neighbor	0.415 [0.399–0.432]	0.204 [0.167–0.241]	2.3×10^{-172}	5.9×10^{-46}	—
PertMean	0.409 [0.376–0.445]	0.254 [0.215–0.292]	1.7×10^{-70}	1.8×10^{-31}	—
LinRidge	0.325 [0.295–0.358]	0.247 [0.209–0.286]	3.6×10^{-80}	3.1×10^{-33}	—
CellFlow	0.310 [0.281–0.343]	0.213 [0.177–0.249]	1.6×10^{-80}	3.5×10^{-45}	—
**Energy distance**					
**GENESIS**	**1.027 [0.972–1.086]**	0.489 [0.460–0.518]	0.34	0.44	★
CtxMean	1.372 [1.185–1.587]	0.371 [0.337–0.405]	2.5×10^{-5}	4.4×10^{-13}	—
Additive	1.373 [1.229–1.533]	0.319 [0.286–0.351]	2.9×10^{-8}	2.7×10^{-25}	—
PertMean	1.438 [1.288–1.606]	0.289 [0.257–0.320]	2.2×10^{-10}	1×10^{-33}	—
LinRidge	1.460 [1.303–1.635]	0.286 [0.254–0.318]	1.4×10^{-10}	2.2×10^{-34}	—
STATE	1.464 [1.377–1.557]	0.206 [0.176–0.235]	6.8×10^{-30}	8.6×10^{-64}	—
PerturbDiff	2.852 [2.675–3.040]	0.033 [0.021–0.045]	4.1×10^{-124}	4.2×10^{-284}	—
CellFlow	13.973 [13.024–14.992]	0.000 [0.000–0.000]	4×10^{-270}	0	—
Nearest-neighbor	16.703 [15.521–17.976]	0.000 [0.000–0.000]	9.7×10^{-275}	0	—

bioRxiv preprint doi: https://doi.org/10.64898/2026.10.30.763081; this version posted November 1, 2026. The copyright holder for this preprint (which was not certified by peer review) is the author/funder, who has granted bioRxiv a license to display the preprint in perpetuity. It is made available under a CC-BY 4.0 International license.

Supplementary Table 2 | GENESIS models trained on reduced data evaluated against experimental reproducibility. Models from Fig. 3a were evaluated across 530 Tahoe-100M plate pairs, scoring predictions against observations from the other plate. Training sets retained fewer perturbed conditions or fewer cells across conditions, with all controls retained. R is the ratio of mean model performance to mean experimental-repeat performance across plates; $R = 1$ indicates equal means. Better model performance corresponds to $R > 1$ for DEG overlap and Pearson Δ, and $R < 1$ for energy distance. Win rate is the fraction of cross-plate predictions outperforming the repeat, with ties counted as half. We report 95% confidence intervals and two-sided p values for $R = 1$ and win rate = 0.5, using the cluster-adjusted tests described in Methods. Stars indicate $p \geq 0.05$ for both tests.

Training data	Perturbed cells	R (95% CI)	Win rate (95% CI)	p (R)	p (win)	Star
**Direction-matched DEG overlap**						
100%	89.5M	0.962 [0.943–0.980]	0.437 [0.408–0.467]	8.6×10^{-5}	3×10^{-5}	—
25% conditions	21.9M	0.967 [0.948–0.986]	0.463 [0.434–0.493]	8×10^{-4}	0.01	—
25% cells	21.9M	0.956 [0.937–0.975]	0.446 [0.417–0.475]	6.9×10^{-6}	2.9×10^{-4}	—
5% conditions	4.6M	0.862 [0.841–0.883]	0.390 [0.358–0.422]	1.4×10^{-29}	3.8×10^{-11}	—
5% cells	4.6M	0.946 [0.927–0.966]	0.444 [0.414–0.474]	2×10^{-7}	2.4×10^{-4}	—
**Pearson Δ**						
100%	89.5M	0.992 [0.975–1.009]	0.479 [0.446–0.513]	0.37	0.22	★
25% conditions	21.9M	1.004 [0.985–1.023]	0.481 [0.447–0.515]	0.71	0.27	★
25% cells	21.9M	1.000 [0.982–1.019]	0.475 [0.440–0.509]	0.99	0.14	★
5% conditions	4.6M	0.924 [0.902–0.946]	0.375 [0.339–0.410]	2.5×10^{-10}	9.5×10^{-12}	—
5% cells	4.6M	1.012 [0.989–1.035]	0.475 [0.439–0.512]	0.31	0.18	★
**Energy distance**						
100%	89.5M	1.028 [0.973–1.087]	0.487 [0.459–0.515]	0.32	0.36	★
25% conditions	21.9M	1.035 [0.978–1.096]	0.493 [0.465–0.521]	0.23	0.64	★
25% cells	21.9M	1.022 [0.967–1.079]	0.484 [0.456–0.511]	0.44	0.25	★
5% conditions	4.6M	1.152 [1.062–1.250]	0.402 [0.373–0.431]	7.1×10^{-4}	1×10^{-10}	—
5% cells	4.6M	1.033 [0.973–1.096]	0.472 [0.442–0.501]	0.29	0.06	★

bioRxiv preprint doi: https://doi.org/10.64898/2026.10.30.763081; this version posted November 1, 2026. The copyright holder for this preprint (which was not certified by peer review) is the author/funder, who has granted bioRxiv a license to display the preprint in perpetuity. It is made available under a CC-BY 4.0 International license.

Supplementary Table 3 | Default GENESIS training settings, as used for the Tahoe-100M benchmark model (Fig. 2). Analysis-specific changes and training durations are described in Methods.

Setting	GENESIS default
**Data**	
Input transformation	log(1+count)
Genes	2,000
Batch (observed cells)	1,792
Loader	scDataset v0.3.0
Block size	1,024 cells
Fetch size	14 minibatches
Sampling	uniform over cells, without replacement
**Architecture**	
Backbone	conditional MLP
Embedding width	256
Hidden widths	4,096–8,192
Activation	GELU
Normalization layers	none
Dropout	none
Noise	uniform
Output activation	ReLU
Output bias	training mean per gene
**Loss**	
Objective	energy score + variogram
Generated cells m	256
Variogram weight	0.5
Variogram exponent p	0.5
Variogram gene pairs per step	6,000
Variogram cells	896 of 1,792 observed; 256 generated each
Variogram estimator	debiased
**Optimization**	
Optimizer	AdamW (β_1 0.9, β_2 0.95)
Peak learning rate	9×10^{-4}
Warm-up	linear, 5% of steps (100–1,000)
LR multipliers	×1, ×0.1 after 20%, ×0.01 after 40% of steps
Final decay	linear to ×0.1 over the last 15% of steps
Weight decay	0.0001
Gradient clipping	none
Precision	bf16 mixed
Seed	64
